# Olfactory and taste dysfunction in COVID-19-incidence and recovery

**DOI:** 10.1186/s43163-023-00383-6

**Published:** 2023-01-23

**Authors:** Surinder K. Singhal, Nitin Gupta, Ravneet R. Verma, Jyotika Sharma, Monali Sah, Shivani Jain, Diksha Kashyap

**Affiliations:** Department of ENT, Head and Neck Surgery, Government Medical College and Hospital, Chandigarh, India

**Keywords:** Olfaction, Smell, Anosmia, Gustatory, Taste, Ageusia, COVID-19

## Abstract

**Background:**

Olfactory and taste dysfunctions have been identified as prominent signs of COVID-19 infection. The data on its prevalence, time of onset, and recovery is highly variable.

**Objective:**

The study was aimed at establishing the incidence of and the factors influencing smell and taste disorders in COVID-19-affected patients.

**Methodology:**

Telephonic interviews were used to collect data on the symptoms of COVID-19-positive patients, with an emphasis on smell and taste disorders. Patients have severe disease and a history of illnesses that may affect olfaction or taste, and those unwilling to participate were excluded.

**Results:**

A total of 1488 COVID-19-positive patients were identified. A total of 772 were included and interviewed, and their data were analysed. A total of 242 (31.3%) patients developed symptoms related to smell and/or taste. Anosmia (149) and ageusia (152) were the most common. Younger patients were more commonly affected (*p* = 0.0016). The presence and degree of smell symptoms and taste symptoms showed a small positive correlation (*r* = .234, *p* < .001). A strong relationship was seen with fever (*r* = .825, *p* < .001) and a significantly moderate relationship with breathing difficulty. There was no significant difference based on age or sex for the recovery of smell or taste sensations. There was a significant correlation between taste recovery and smell recovery times (*p* < 0.00001).

**Conclusion:**

Smell and taste disorders, as early clinical symptoms of COVID-19, may have a diagnostic as well as a prognostic value. Treatment protocols for these patients are yet to be defined. A positive association between these symptoms and breathing difficulty was found, and we recommend COVID-19 testing and monitoring of symptoms for all patients with new-onset OTD symptoms. A combination of active enquiry about these symptoms, along with objective testing when the patients present with COVID-19 symptoms may help in better understanding of the pathophysiology and timely initiation of treatment.

## Background

The clinical presentation among COVID-19 patients has ranged from asymptomatic disease to life-threatening complications. There is growing evidence that olfactory and taste dysfunctions (OTD) are prominent signs of COVID-19 infection. These symptoms may occur alone or accompanied by other symptoms [1, 2].

The variation in presentation for smell and taste symptoms may have a genetic predisposition. It was hypothesised that this is due to the differences in the binding affinity of the ACE2 receptor for the virus [3]. The incidence and recovery thereof, from these symptoms, are variable across the literature. This study was conducted to help us identify the patterns of symptomatology, at-risk populations, and frequency of temporary vs permanent loss of special senses.

## Methods

Adult patients (≥ 18 years) diagnosed with COVID-19 between February 2021 and November 2021 were contacted telephonically. All included patients had tested positive on RT-PCR testing. The Institutional Research Committee and the Institutional Ethical Core Committee approval was obtained (GMCH/IEC/2020/413/194). Telephonic informed consent was taken from every patient. Patients who had developed breathing difficulty/needed intubation/ICU admission or had contracted COVID-19 more than once and those with a history of diabetes mellitus, auto-immune disorders or any neuropathies, hearing or speech disability, cognitive disability or psychiatric disorder were excluded from the study. Pregnancy at the time of contracting the virus was also an exclusion criterion.

Patients were asked about the changes in sensation of smell and/or taste which developed at or around the time of testing positive for COVID-19. Those symptoms were questioned about the onset and the duration of OTD symptoms and other COVID-19 symptoms. They were followed up telephonically, till the taste and smell had completely recovered or up to 4 weeks from the date of COVID-19 testing, whichever was earlier.

Continuous variables were expressed as mean and standard deviation (SD) and compared with Student’s *t*-test. Categorical variables were expressed as number and percentage (%) and compared by the chi-square test. The correlation was calculated by Pearson’s (nominal) and Spearman’s (ordinal) rank correlation coefficient. A *p*-value < 0.05 was considered significant.

## Results

Every record of a COVID-19 patient who tested positive at the hospital was screened, and a total of 1488 phone numbers were extracted. A few phone numbers were incorrect or not reachable, some patients were not available or unwilling to take part in the study. A total of 954 patients agreed to take part in the study, and data was collected. A total of 182 were excluded as per the exclusion criteria. Data for 772 patients were eventually analysed.

**Fig. 1:**
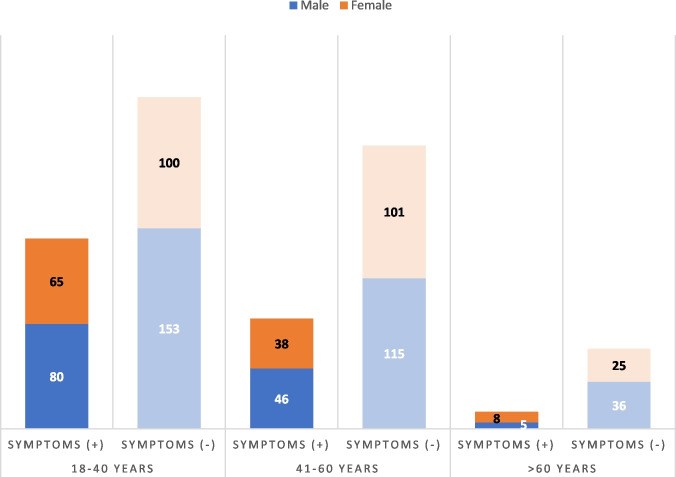
Age and sex distribution of patients with and without OTD symptoms

The male-to-female ratio was 1.29 (435 male patients vs 337 female). The patients were divided into 3 broad age categories: 18 to 40 years (398 patients), 41 to 60 years (300 patients), and over 60 years (74 patients) (Fig. 1).

Of the 772 patients, 242 (31.3%) patients developed OTD symptoms. Taste/gustatory dysfunction (GD) was noted in 27% (209), and 25.5% (197) had smell/olfactory dysfunction (OD). Anosmia (149) and ageusia (152) were more commonly reported than hyposmia (44) and hypogeusia (44) (Table 1). GD without OD was seen in 45 patients, and 33 patients had OD without GD (Table 2).

**Table 1:** Categorization of smell and taste abnormalities in our study population

	**Number of patients (percentage)**
**Dysfunction of smell**	
Complete loss (anosmia)	149 (19.3%)
Partial loss (hyposmia)	44 (5.7%)
Altered sensation (parosmia)	4 (0.5%)
**Dysfunction of taste**	
Complete loss (ageusia)	152 (19.7%)
Partial loss (hypogeusia)	44 (5.7%)
Altered sensation (parageusia)	13 (1.7%)

**Table 2:** Number of patients with dysfunction of smell and/or taste

	Total	Male	Female	
Total patients evaluated	772	435	337	
Without OTD	530	304	226	***p* = 0.4**
With OTD	242	131	111	
Total OD (with or without GD)	197	102	95	
Total GD (with or without OD)	209	115	94	
With both OD and GD	164	86	78	***p* = 0.28**
Only OD	33	16	17	
Only GD	45	29	16	

There was no significant predisposition of OTD symptoms towards either sex. A significantly higher number of younger patients (36.4%) had OTD symptoms (*p* = 0.0016) (Table 3).

**Table 3:** Age-wise distribution of olfactory and gustatory dysfunction

	Total	18–40 years	41–60 years	> 60 years	
Without OTD	530	253	216	61	***p* = 0.0016**
With OTD	242	145	84	13	
Total OD (with or without GD)	197	124	64	9	
Total GD (with or without OD)	209	125	71	13	
With both OD and GD	164	104	51	9	***p* = 0.16**
Only OD	45	21	20	4	
Only GD	33	20	13	0	

The presence and degree of OD and GD showed a significant small positive correlation (*r* = 0.234, *p* < 0.001). There was a significant large positive relationship between OTD and fever, (*r* = 0.825, *p* < 0.001) and a significant moderate positive relationship between OTD and breathing difficulty (*r* = 0.342, *p* < 0.001).

Of the 242 patients with OTD, the majority (154) had fever as the first symptom, followed by malaise (Table 4). OTD was the first symptom in 33 patients. Among others, OD and GD developed within the first 3 days of the primary symptom in 67% and 65% of the patients, respectively.

**Table 4:** Symptoms of patients at the time of presentation

1st symptom	No. of patients
Fever	154
Malaise	27
Taste disturbance	16
Smell disturbance	15
Smell and taste disturbance	2
Nasal block/discharge	7
Breathlessness	4
Cough	5

OD presented after a mean duration of 2.59 days (SD = 1.89) and GD after a mean of 2.83 days (SD = 3.45). There was no significant difference between the two (*p* = 0.19). Most patients recovered smell/taste within the follow-up period. Out of 197 with OD, 2 had partial and 193 had full recovery. Among those with GD (209), 200 had complete recovery while 3 had partial recovery. Persistent smell and taste disturbance at the end of the follow-up period was seen in 4 and 9 patients, respectively. There was no significant difference in the time taken from onset to start of recovery and to complete recovery (Table 5).

**Table 5:** Time taken from onset to beginning of recovery and to complete recovery

Recovery	Dysfunction	Number of patients (*n*)	Number of days, mean (standard deviation)	*p* value
Number of days from onset to start of recovery	Taste	203	10.1 (11.75)	**0.91**
	Smell	195	10.2 (14.24)	
Number of days from the start of recovery to complete recovery	Taste	200	15.6 (20.27)	**0.42**
	Smell	193	6.87 (15.27)	
Number of days from onset to complete recovery	Taste	200	15.56(20.063)	**0.58**
	Smell	193	16.87 (26.65)	

**Fig. 2:**
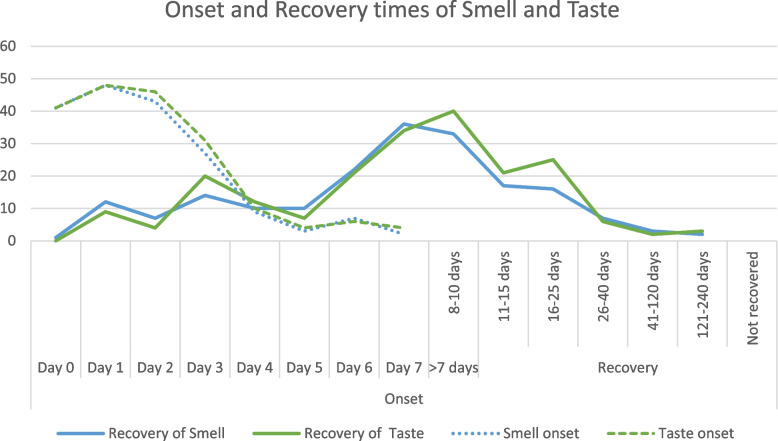
Representation of the onset of OTD symptoms and their recovery over time

Recovery from OTD was compared for age, sex, and symptom at presentation, showing no significant differences between the groups (Table 6). While there was a significant correlation between taste and smell onset and recovery times (Fig. 2), there was a non-significant, very small, positive relationship between the recovery duration of OTD and the recovery from other symptoms (Table 7).

**Table 6:** Comparison of the effect of age, sex, and symptom at presentation on the duration of recovery

			Sample size	Number of days, mean (standard deviation)	Test	Association	*p* value
Smell recovery	Sex	Male	101	14.58 (25.23)	*t*-test	*t* = − 1.25	*p* = 0.21
		Female	92	19.38 (28.05)			
	Age (years)	18–40	123	10.83 (16.2)	*t*-test	*t* = 1.13	*p* = 0.26
		> 40	67	13.5 (14.28)			
Taste recovery	Sex	Male	113	13.84 (18.66)	*t*-test	*t* = − 1.35	*p* = 0.18
		Female	88	17.68 (21.55)			
	Age (years)	18–40	121	16.82 (24.11)	*t*-test	*t* = 1.18	*p* = 0.24
		> 40	81	13.46 (11.15)			
Smell recovery	1st symptom	OTD	26	11.5 (8.92)	*t*-test	*t* = 1.12	*p* = 0.26
		Others	162	17.9 (28.76)			
Taste recovery	1st symptom	OTD	24	11.04 (8.6)	*t*-test	*t* = 1.16	*p* = 0.25
		Others	178	16.07 (20.99)

**Table 7:** Correlation of recovery time of OTD symptoms with each other and other common symptoms

	**Recovery time**	**No. of patients (*n*)**	**Test**	**Strength of association**	**Significance**
Co-relation of recovery time	Smell and taste	160	Pearson correlation coefficient	*r* = 0.91	*p* < 0.00001
	Smell and fever	156		*r* = 0.003	*p* = 0.97
	Taste and fever	169		*r* = 0.02	*p* = 0.84
	Smell and breathing	29		*r* = − 0.08	*p* = 0.69
	Taste and breathing	34		*r* = 0.29	*p* = 0.09

OTD and early (≤ 15 days) vs late (> 15 days) recovery from the symptoms were analysed to any correlation with other symptoms like fever, cough, malaise, or breathing difficulty. These symptoms were not significant predictors for OTD symptoms or their recovery time (*p* = 0.94).

## Discussion

There is sufficient evidence to confirm that olfactory and taste dysfunctions are prominent signs of COVID-19 infection. These symptoms can range from total loss (anosmia and ageusia) or partial loss (hyposmia and hypogeusia) to abnormal perception (parosmia and parageusia) and perception without stimulus (phantosmia and phantogeusia) [4].

### Prevalence

Using an advanced deep neural network, the prevalence of anosmia/dysgeusia was determined to be 27.1 times in patients with COVID-19. OTD was identified as one of the most salient early features of COVID-19 infection [5]. The frequency of reported symptoms though has varied widely across the available literature. The prevalence of OD ranges from 1.6 to 98.33%. The highest mean prevalence was recorded in the Americas (56.05%), Europe (55.44%), and the Middle East (50.42%), while in Asia and Africa, it was 28.83% and 26.5%, respectively. The prevalence of GD ranges from 1.2 to 90.3%. The mean prevalence was higher in Europe (58.6%) and the Americas (52.53%) and much lower in Asia (27.19%), the Middle East (23.23%) and Africa (22.1%) [6].

While some studies report a similar prevalence of both taste and smell disorders, others have shown greater variations. This may be due to the heterogeneous study designs and the diverse and subjective nature of the complaints. Studies have shown inconsistent results with regard to which sensation is affected more or if the absence of sensation is more common than reduced sensation.

The pooled proportion of patients with OD and GD was 41% and 38.2%, respectively, in one meta-analysis [7]. Similar degrees of loss of smell (65.3%) and taste (64.3%) were reported by Alshakhs et al [8]. OD and GD in over 80% and 90% of patients, respectively, were reported by Beltrán-Corbellini et al [9]. On the other hand, in a large study of over 2000 patients using online questionnaires, 87% of patients had OD and 56% suffered from GD [10].

Klopfenstein et al. stated that 47% of their patients had anosmia and 85% had dysgeusia [11].

In a single-centre study, 41.7% of patients reported combined disorders of olfaction and taste, isolated OD was seen in 12.5%, and isolated GD in 14.4% [12]. In a multicentre study, the authors found 79.3% of patients suffering from combined disturbances, but only 8.6% and 12.1% had isolated OD and GD, respectively [13].

Catton and Gardner observed that smell loss in the absence of taste loss was more prevalent which is supported by Lee et al. (36% more cases of smell loss without taste loss) [14, 15]. In our study, 31.3% of patients developed symptoms related to smell or taste dysfunction. Twenty-seven per cent reported GD, and 25.5% had OD. A higher proportion of patients had GD without OD than the other way around (21.53% vs 16.75%). Anosmia (19.3%) and ageusia (19.7%) were more common than partial loss (5.7% for both).

Lechien et al. reported that 85.6% of their patients had OD. Among them, 79.6% of patients were anosmic, and 20.4% had hyposmia. GD was reported in 88% of their patients [1]. Out of 180 patients evaluated by Tipirdamaz et al., 78.6% had anosmia, and 21.4% had hyposmia [16].

Hyposmia (52.2%) and hypogeusia (34.5%) were more common compared to anosmia (17.7%) and ageusia (10.4%) in a study of 345 patients by Vaira et al. They also determined that in the early days of the infection, severe dysfunctions affected 70.9% of patients while in the later part (after day 10) most of the symptoms were mild to moderate [13].

### First symptom

OTD was the first symptom in 29.2% and the only one in 9.5% of the cases in a multicentric study [13]. In another study, OD appeared before the other symptoms in 11.8% of cases [1].

18.1% of patients had OTD at the time of presentation in a single-centre study [12]. OTD represented the first clinical sign of the disease in 13.6% of our patients.

### Age and sex

While no significant association with age and sex was reported by Varia et al., other studies found OTD to be significantly more common in younger and female patients [13, 11, 17]. Hafez et al. reported that females had a significantly higher prevalence of OTD (*p*< 0.001), but there was no association with age [18]. The higher incidence of OTD in women has been attributed to gender differences in the inflammatory response process [19]. Contrary to their findings, our study population showed a significant association with younger age but no association with sex. Although some studies do suggest a correlation, one systematic review found no association between sensory recovery and age [20].

### Objective testing vs subjective reporting

Thirty-eight per cent (38%) of patients with self-reported symptoms in one study had normal smell on testing and suggested that the prevalence of these complaints would be overestimated in studies based on subjective reports [21]. Vaira et al. on the other hand found mild hyposmia in 10.7% of patients who reported no OD (*p* = 0.000) and 70% of patients who reported complete resolution, on testing proved to be hyposmic (*p* = 0.000). Similarly, among those who reported complete taste recovery, hypogeusia was detected in 28.8%. Patients who reported isolated dysfunctions of either taste or smell, on testing, were found to be hyposmic in 32.3% (*p* = 0.000) and hypogeusic in 22.7% (*p*= 0.024) of the cases, leading the authors to conclude that interview studies may be underestimating the frequency of these disorders [13].

### Association between symptoms

Attempts have been made to quantify the relationship between olfactory and taste symptoms. Lechien et al. and Ciofalo et al. reported a significant relationship between the two (*r* = 0.91) (*p*< 0.0001) [1, 22]. Ali et al. and Catton and Gardner found a moderate correlation between the intensity of the two symptoms, while a weak correlation was demonstrated by Song et al. in a larger study population (*n*= 1172) [14, 23].

The ANOSVID Study found GD to persist more often in patients with persistent olfactory symptoms (*p*< 0.001). Asthma and cacosmia were also significantly linked with the persistence of olfactory dysfunction [16]. Our study population showed a significant small positive relationship between OD and GD (*r* = 0.234, *p* < 0.001). There was a strong positive relationship between OTD with fever and a moderate relationship with breathing difficulty.

### Effects on quality of life (QOL)

OTD affects the QOL of patients considerably, even in the absence of other symptoms. Persistent dysfunction can worsen patient well-being and may even lead to psychiatric disorders such as depression and anxiety [6].

A study evaluating mental health responses to OTD reported that more than 40% of their patients experienced that loss of smell made them feel isolated, 2/3rd of them had a change in dietary habits and over 1/3rd found it difficult to take part in normal daily activities. Anosmia was a trigger for loss of temper in 28.2% and dysgeusia caused an imbalance in the life of 62.1% of the patients [8].

### Recovery

After 2 weeks of testing positive, the proportion of recovered patients ranged from 25.5 to 80% [1, 11]. In a retrospective analysis using an online survey, full recovery was calculated to have a median duration of 12.5 days, with about half the patients recovering in that time [24].

Another study from Israel reported a median recovery from anosmia of 7.6 days (35.7% recovered) [25].

Lee et al. noted that it took over 3 weeks for a cohort of 3191 patients in South Korea to fully recover. The younger patients (ages 20–39) were more likely to have delayed recovery from anosmia [26].

Persistent OTD, even 7 months after the symptom onset, was present in 24% of patients in the study by Nguyen et al. and was more common in women (73.3%) than in men (26.7%) [27].

Even after 5 weeks, OD and GD continued in 37% and 7% of patients, respectively, in a smaller study sample (*n*= 72) [28]. One multicentre study also reported about 56% of patients having persistent OD even after the resolution of the other symtpoms [1].

Cecchetto et al. reported a strong relationship between the recovery of smell and taste [29]. Another study suggested that the association of recovery time was stronger than the correlation between the severity of these symptoms [14].

In our cohort, the mean duration for the recovery of taste was 15.56 days and 16.87 days for smell recovery. We found a strong, significant correlation between the recovery times of OTD and fever while only a weak and statistically insignificant correlation existed with recovery time and breathing difficulty.

In the ANOSVID Study, 85% of patients recovered from OD within 3 months, followed by a recurrence after recovery, resulting in 41% of cases having OD after more than 9 months [16].

### Pathophysiology

ACE2 receptors are located on the olfactory and respiratory epithelium of the nasal cavity and even on the olfactory bulb [30]. Primarily located in the motile cilia, ACE2 receptors are thought to be the site of COVID-19 viral entry [31].

OD in COVID-19 is thought to be due to damage to the supporting cells of the olfactory epithelium which have an abundance of ACE2 receptors. The virus attaches to these receptors and induces cell death, leaving sensory neurons vulnerable and without nutrients [32].

Direct infection of taste cells causing cell death or reduced secretion of neurotransmitters has been proposed as a cause for altered taste sensation [33, 34].

Viral binding to ACE2 may also lead to GD since the renin–angiotensin–aldosterone system is believed to take part in the cleaving of gustatory molecules [6].

GD and its improvement have been linked with the blood levels of IL-6 as well [35].

### Clinical utility

A strong association has been established between chemosensory dysfunctions of smell/taste and a positive COVID-19 test. Therefore, it may even serve as a preliminary tool for identifying and isolating suspected cases. OTD can help to predict clinical outcomes. Quicker recovery can indicate the resolution of viral infection [36].

### Limitations

Our study is a telephonic, subjective, self-reported assessment which is affected by recall bias, lack of accurate grading, and differentiation of true dysfunction or loss. This may lead to imprecise (over or under) reporting. Although we did try to evaluate the use of pharmaceutical interventions that can alter the progression of OTD, most patients were not able to recollect information about the medications consumed. Because there were no control groups, we could not compare the chemosensory dysfunction to its prevalence in the general population.

## Conclusion

Smell and taste disorders, as early clinical symptoms of COVID-19, may have a diagnostic as well as a prognostic value. They may precede the onset of full-blown clinical disease. The recovery of these symptoms may also indicate recuperation from the infection. A consensus on factors contributing to the type of dysfunction, time of onset, speed of recovery, and long-term effects is lacking at present. In the published literature, the prevalence of OTD has ranged considerably and so has the relationship of these symptoms with other COVID-19 symptoms, patient demographics, as well as objective evaluation of the symptoms. Persistent OTD is seen in fewer patients but remains a matter of concern. We found a positive association between OTD symptoms and the development of breathing difficulty; therefore, COVID-19 testing and monitoring of symptoms for all patients with new onset OTD symptoms are recommended. A combination of active enquiry about these symptoms, along with objective testing when the patients present with COVID-19 symptoms may help in better understanding of the pathophysiology and timely initiation of treatment.

## Data Availability

The datasets used and/or analysed during the current study are available from the corresponding author upon reasonable request.

## Abbreviations

OTD: Olfactory and taste dysfunction
SD: Standard deviation
GD: Taste/gustatory dysfunction
OD: Smell/olfactory dysfunction
